# Computational Redesign of Bacterial Biotin Carboxylase Inhibitors Using Structure-Based Virtual Screening of Combinatorial Libraries

**DOI:** 10.3390/molecules19044021

**Published:** 2014-04-02

**Authors:** Michal Brylinski, Grover L. Waldrop

**Affiliations:** 1 Division of Biochemistry and Molecular Biology, Louisiana State University, Baton Rouge, LA 70803, USA; E-Mail: gwaldro@lsu.edu; 2 Center for Computation & Technology, Louisiana State University, Baton Rouge, LA 70803, USA

**Keywords:** biotin carboxylase, acetyl-CoA carboxylase, biotin carboxylase inhibitors, amino-oxazole, combinatorial chemistry, cheminformatics, ligand docking, virtual screening, *e*SimDock

## Abstract

As the spread of antibiotic resistant bacteria steadily increases, there is an urgent need for new antibacterial agents. Because fatty acid synthesis is only used for membrane biogenesis in bacteria, the enzymes in this pathway are attractive targets for antibacterial agent development. Acetyl-CoA carboxylase catalyzes the committed and regulated step in fatty acid synthesis. In bacteria, the enzyme is composed of three distinct protein components: biotin carboxylase, biotin carboxyl carrier protein, and carboxyltransferase. Fragment-based screening revealed that amino-oxazole inhibits biotin carboxylase activity and also exhibits antibacterial activity against Gram-negative organisms. In this report, we redesigned previously identified lead inhibitors to expand the spectrum of bacteria sensitive to the amino-oxazole derivatives by including Gram-positive species. Using 9,411 small organic building blocks, we constructed a diverse combinatorial library of 1.2 × 10^8^ amino-oxazole derivatives. A subset of 9 × 10^6^ of these compounds were subjected to structure-based virtual screening against seven biotin carboxylase isoforms using similarity-based docking by *e*SimDock. Potentially broad-spectrum antibiotic candidates were selected based on the consensus ranking by several scoring functions including non-linear statistical models implemented in *e*SimDock and traditional molecular mechanics force fields. The analysis of binding poses of the top-ranked compounds docked to biotin carboxylase isoforms suggests that: (1) binding of the amino-oxazole anchor is stabilized by a network of hydrogen bonds to residues 201, 202 and 204; (2) halogenated aromatic moieties attached to the amino-oxazole scaffold enhance interactions with a hydrophobic pocket formed by residues 157, 169, 171 and 203; and (3) larger substituents reach deeper into the binding pocket to form additional hydrogen bonds with the side chains of residues 209 and 233. These structural insights into drug-biotin carboxylase interactions will be tested experimentally in *in vitro* and *in vivo* systems to increase the potency of amino-oxazole inhibitors towards both Gram-negative as well as Gram-positive species.

## 1. Introduction

The dramatic increase in the number of pathogenic bacteria with extensive resistance to antibiotics has been well documented in both the scientific literature [1,2] and popular media. For instance, resistance is particularly problematic in the Gram-positive organism *Staphylococcus aureus* (e.g., methicillin resistant *Staphylococcus aureus*-MRSA) as well as a number of Gram-negative organisms like *Klebsiella pneumonia*, *Acinetobacter baumannii*, and *Pseudomonas aeruginosa* [3]. In order to mitigate this problem, new antibiotics directed against new target molecules are desperately needed. Since fatty acids are only used for membrane biogenesis in bacteria, the enzymes of the fatty acid biosynthetic pathway are potential targets for the development of novel antibacterial agents [4,5,6].

**Scheme 1:**
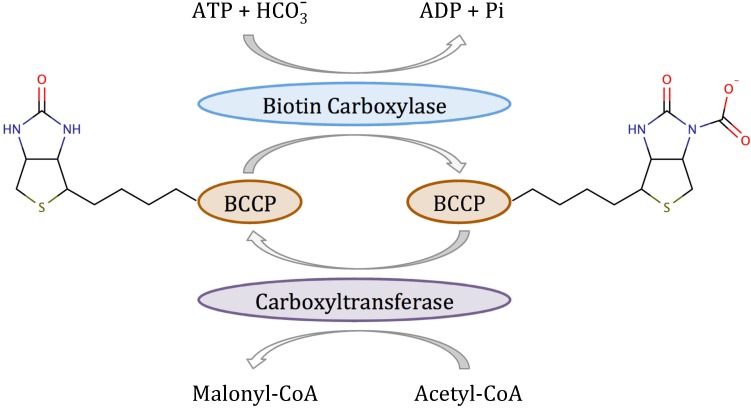
Reaction mechanism of bacterial acetyl-CoA carboxylase.

The rate-determining and committed reaction in fatty acid biosynthesis in bacteria is catalyzed by acetyl-CoA carboxylase [7]. Acetyl-CoA carboxylase (ACC) is a multifunctional enzyme that catalyzes the two-step reaction shown in Scheme 1 [8]. In the first half-reaction, biotin carboxylase (BC) catalyzes the ATP-dependent carboxylation of the vitamin biotin, which *in vivo* is covalently attached to the biotin carboxyl carrier protein (BCCP). In the second half-reaction, carboxyltransferase catalyzes the transfer of the carboxyl group from biotin to acetyl-CoA to form malonyl-CoA, which is the substrate for fatty acid synthase. In Gram-positive and Gram-negative bacteria, BC, BCCP and carboxyltransferase are separate proteins that form a complex *in vivo* [9]. However, when either BC or carboxyltransferase are purified, they retain their enzymatic activity in the absence of the other two components. Most importantly, both BC [10] and carboxyltransferase [11] have been validated as targets for antibacterial development.

Three different classes of molecules have been found to inhibit bacterial BC and also exhibit antibacterial activity: pyridopyrimidines [10], amino-oxazoles [12] and the benzimidazole carboxamides [13]. Scientists at Pfizer were the first to discover an antibiotic targeting BC [10]. Whole cell screening of a 1.6 × 10^6^ compound library revealed that pyridopyrimidines had potent antibacterial activity. When strains of *H*. *influenzae* resistant to the pyridopyrimidines were generated, the resistant mutation mapped to the gene coding for BC. The pyridopyrimidines inhibited BC with a *K*_i_ of 0.8 nM by competing with ATP for binding to the enzyme. Surprisingly, and fortunately, the pyridopyrimidines did not inhibit human acetyl-CoA carboxylase. However, the pyridopyrimidines were only effective against Gram-negative organisms such as *E*. *coli*, *H*. *influenzae* and *M*. *catarrhalis*, and showed limited antibacterial activity against Gram-positive organisms.

**Figure 1:**
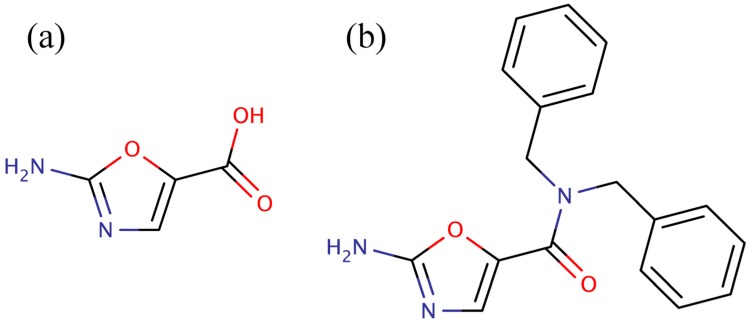
Low molecular weight inhibitors of biotin carboxylase. (**a**) 2-Amino-oxazole, (**b**) dibenzylamide analog.

Using the three-dimensional structure of BC bound to pyridopyrimidines as a starting point, the Pfizer group then applied a combination of virtual screening and fragment based drug design to discover a series of low molecular weight inhibitors of BC [12,14]. The advantage of these low molecular weight inhibitors *versus* the pyridopyrimidines is that they were more amenable to synthetic elaboration. One of these inhibitors, 2-amino-oxazole (Figure 1a), was subjected to fragment growing to generate the dibenzylamide analog shown in Figure 1b. Like the pyridopyrimidines, the dibenzylamide analog inhibited bacterial BC by binding in the ATP binding site, but did not inhibit the human enzyme. Also, like the pyridopyrimidines, amino-oxazole dibenzylamide showed strong antibacterial activity against Gram-negative organisms, while exhibiting limited activity against Gram-positive organisms. Thus, the major shortcoming of both the pyridopyrimidines and the amino-oxazole derivatives as antibiotics is that they had a very narrow spectrum of activity, *i.e.*, they were only effective against Gram-negative bacteria. Since the pyridopyrimidines are not synthetically tractable, the best chance for developing a broad-spectrum antibacterial agent that targets BC is to focus on the amino-oxazole scaffold. While the amino-oxazole fragment (Figure 1a) can serve as an anchor to bind in the ATP binding site, the carboxyl group provides a very accessible functionality that can be easily modified with a variety of nitrogen containing ligands using standard peptide coupling conditions. This was the synthetic approach used by the Pfizer group in their initial studies of amino-oxazole derivatives [12,14]. Therefore, a fragment could be attached to amino-oxazole that renders the molecule able to bind to BC from both Gram-negative and Gram-positive bacteria would have the potential to exhibit broad-spectrum activity. Thus, the purpose of this study is to identify low molecular weight fragments that could be coupled to the amino-oxazole scaffold and enable the molecule to bind to BC from both Gram-negative and Gram-positive bacteria. The major hurdle is how to identify low molecular weight fragments that can bind to BC from both Gram-negative and Gram-positive bacteria.

Computer-based approaches are routinely used in modern drug discovery to significantly reduce time and costs associated with the development of new biopharmaceuticals. Many experimental techniques, such as high-throughput screening and combinatorial chemistry, involve relatively random processes, thus the overall efficiency of the discovery process can be greatly improved by using computer technologies to design more focused experiments [15]. Amongst many computational methods, structure-based virtual screening is one of the most widely used to support drug development [16,17]. These algorithms extensively use structural information available for target proteins to limit the size of chemical libraries to those compounds that are most likely to exhibit the desired bioactivities. In virtual screening by molecular docking, each drug candidate is docked into the protein target using a conformational search algorithm and a scoring function, which is followed by affinity prediction from drug-target interactions modeled at the molecular level. Considering the constantly increasing throughput capabilities of high-performance computing systems, structure-based virtual screening can be applied to systematically evaluate a large number of small organic compounds prior to experimental testing [18]. On that account, these techniques are particularly powerful in investigating diverse combinatorial libraries, whose considerable size in the order of millions of molecules surpasses the capacity of experimental high-throughput screening. Examples of structure-based virtual screening include the successful development of anti-influenza agents [19], the discovery of novel compounds with anti-herpes activity [20], and the discovery of a novel high-affinity ligand for human carbonic anhydrase II [21].

In this study, we first constructed a large combinatorial library of antibiotic candidates containing the amino-oxazole scaffold. These compounds were subsequently subjected to structure-based virtual screening against several BC isoforms from both Gram-positive and Gram-negative species. Binding poses of potentially broad-spectrum inhibitors selected from docking simulations were analyzed in order to shed light onto the possible structural determinants responsible for the high antibiotic potency of amino-oxazoles towards bacterial BC.

## 2. Results and Discussion

**Figure 2:**
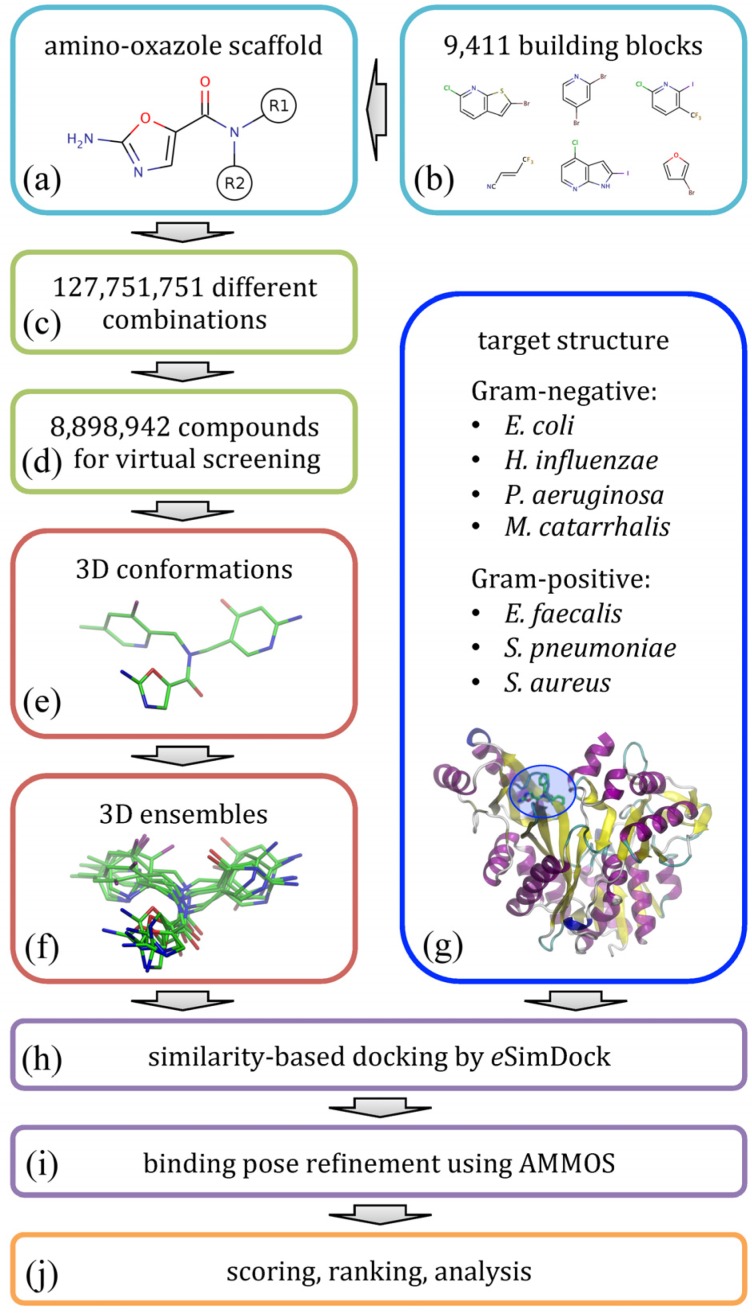
Flowchart of the computational redesign of biotin carboxylase inhibitors. Amino-oxazole scaffold (**a**) is used to anchor different chemical moieties (**b**) at positions R1 and R2 in order to construct a large combinatorial library of amino-oxazole derivatives (**c**). For a subset of compounds (**d**), 3D conformations (**e**) and conformational ensembles (**f**) are generated. These are systematically docked to biotin carboxylase isoforms from seven bacterial species (**g**) using *e*SimDock (**h**). Initial binding poses are subject to all-atom refinement using AMMOS (**i**). The final binding poses are scored, ranked and analyzed (**j**).

The flowchart shown in Figure 2 illustrates the modeling procedure used to redesign amino-oxazole inhibitors of BC. Specifically, our goal is to find chemical moieties which, when attached at positions R1 and R2 of the amino-oxazole scaffold (Figure 2a), would result in an increased potency of this class of BC inhibitors against both Gram-negative and Gram-positive bacteria species. 

Using a library of small organic building blocks (Figure 2b) and virtual combinatorial chemistry techniques, we systematically explored all possible combinations of small fragments attached at positions R1 and R2 generating a non-redundant dataset of nearly 1.3 × 10^8^ amino-oxazole derivatives (Figure 2c). This number of molecules is too large for structure-based virtual screening, therefore we randomly selected a subset of 8.9 × 10^6^ compounds for subsequent modeling stages (Figure 2d). In order to perform molecular docking simulations, for each library molecule, we first generated its three-dimensional representation (Figure 2e) and then a non-redundant ensemble of low-energy conformations (Figure 2f). Docking simulations were performed against BC structures from seven bacterial species (Figure 2g); these structures were constructed by mutating binding site residues in the crystal structure of *E. coli* enzyme according to a multiple sequence alignment of BC isoforms. Structure-based virtual screening of amino-oxazole derivatives was carried out using *e*SimDock [22] against all tested BC isoforms (Figure 2h). *e*SimDock is a pseudo-flexible docking approach, which systematically explores all low-energy conformations in the docking ensemble using a rigid-body optimization of protein-ligand interactions. In practice, our virtual screening protocol constructed, optimized and scored ca. 3.1 × 10^9^ three-dimensional models of drug-target complexes. Subsequently, the top-scored conformations selected from individual docking simulations were subjected to all-atom refinement using molecular mechanics (Figure 2i). Finally, based on the predicted binding affinity, the energy of molecular interactions, and other scores collected from docking and refinement simulations, we ranked the library of amino-oxazole derivatives, selected promising inhibitor candidates, and performed a detailed analysis of modeled protein-ligand interactions (Figure 2j).

### 2.1. Isoforms of Biotin Carboxylase

Known BC inhibitors, including those based on the amino-oxazole scaffold, target the enzyme’s ATP binding site. This region of the BC structure, composed of about 20 amino acid residues listed in Table 1, is highly conserved across isoforms from different bacterial species [23]. In order to quantify the amino acid variability at a given position in the various BC isoforms we calculated the Shannon entropy, which provides a simple measure of uncertainty in a data set [24]. The Shannon entropy was determined from sequence profiles generated by PSI-BLAST [25] for *E*. *coli* BC against a non-redundant collection of protein sequences from the Reference Sequence database (RefSeq) [26]. The maximum entropy calculated for a generic protein-like composition according to amino acid frequencies provided by UniProtKB/Swiss-Prot [27] is 4.19 bits. The average ± standard deviation entropy over the entire BC sequence and binding site residues is only 2.24 ± 0.80 and 1.41 ± 0.76 bits, respectively, indicating the residues forming the ATP binding site in BC are indeed highly conserved. Nevertheless, some residue positions, e.g., 157, 163, 202, 203, and 438, exhibit noticeable sequence variability (residue numbers in this paper are given according to the sequence of *E. coli* BC). Next, we used *e*FindSite [28] to calculate the probability and confidence of ligand binding for residues within the ATP binding site. The primary application of *e*FindSite is binding pocket prediction, however, it can also be used to examine known binding pockets by analyzing ligand-binding patterns across sets of closely as well as remotely related proteins. The residues in BC isoforms from Gram-negative and Gram-positive species found to be important for ligand binding are listed in Table 1. Several residues, e.g., 131, 157, 159, 201-204, 278, and 287-288, are assigned a high ligand-binding probability, which shows that these positions often form direct interactions with small molecules in close and remote homologues of BC. While many of these are absolutely conserved, e.g., K116, V131, K159, G163-166, E201, Q233, and E276, some positions are consistently different in Gram-negative (M169, L204, I287, I437) and Gram-positive species (I169, I204, M287, T437). These subtle sequence differences are particularly important in designing broad-spectrum BC inhibitors, which need to exhibit a certain level of promiscuity to target the binding sites of BC from both Gram-positive and Gram-negative bacteria.

**Table 1:** Binding site residues of biotin carboxylase from Gram-negative and Gram-positive bacteria species.

Residue number	Sequence entropy ^a^	*e*FindSite ^b^		Sequence, Gram-negative ^c^				Sequence, Gram-positive ^d^		
		Probability	Confidence	*Ec*	*Hi*	*Pa*	*Mc*	*Ef*	*Sp*	*Sa*
116	0.747	0.687	0.211	K	K	K	K	K	K	K
131	1.917	0.802	0.452	V	V	V	V	V	V	V
157	2.003	0.710	0.373	I	I	I	I	M	I	I
159	0.763	0.733	0.403	K	K	K	K	K	K	K
163	2.153	0.304	0.159	G	G	G	G	G	G	G
164	1.289	0.463	0.202	G	G	G	G	G	G	G
165	0.659	0.553	0.318	G	G	G	G	G	G	G
166	0.020	0.599	0.355	G	G	G	G	G	G	G
169	1.861	0.456	0.195	M	M	M	M	I	I	I
201	1.941	0.870	0.567	E	E	E	E	E	E	E
202	2.096	0.869	0.419	K	K	K	R	K	R	K
203	2.183	0.864	0.457	Y	Y	F	F	I	V	F
204	1.939	0.874	0.660	L	L	L	L	I	I	I
233	1.130	0.368	0.139	Q	Q	Q	Q	Q	Q	Q
236	1.924	0.569	0.369	H	H	H	H	N	N	M
276	0.786	0.553	0.396	E	E	E	E	E	E	E
278	1.994	0.761	0.608	L	L	L	L	L	L	I
287	1.137	0.823	0.821	I	I	I	I	M	M	M
288	0.001	0.755	0.645	E	E	E	E	E	E	E
437	0.491	0.257	0.174	I	I	I	I	T	T	T
438	2.586	0.071	0.108	H	H	H	H	S	S	N

^a^ Calculated from sequence profiles generated by PSI-BLAST. ^b^ Probability and confidence of ligand binding estimated by *e*FindSite. ^c^ Ec — *E. coli*, Hi — *H. influenzae*, Pa — *P. aeruginosa*, Mc — *M. catarrhalis*. ^d^ Ef — *E. faecalis*, Sp — *S. pneumoniae*, Sa — *S. aureus*.

Three-dimensional models of BC isoforms from *H. influenzae*, *P. aeruginosa*, *M. catarrhalis*, *E. faecalis*, *S. pneumoniae*, and *S. aureus* were constructed using homology modeling based on the *E. coli* enzyme. Using the crystal structures of *P. aeruginosa* (PDB-ID: 2vqd) and *S. aureus* strains (PDB-ID: 2vpq), we estimate that the backbone Cα-RMSD of these models is ~1 Å (0.93 Å and 1.02 Å for 2vqd and 2vpq, respectively). Furthermore, the heavy-atom RMSD calculated over the ATP binding site in the *P. aeruginosa* and *S. aureus* BC isoforms is only 1.04 Å and 1.28 Å, respectively. We note that the ligand docking approach used in this study, *e*SimDock, was specifically designed to tolerate structural imperfections in modeled protein structures, up to 3–5 Å Cα-RMSD [22], thus the quality of BC models is sufficient for their application in structure-based virtual screening.

### 2.2. Molecular Docking Benchmarks

Ligand docking is a critical component of our virtual screening protocol. It is known that the accuracy of docking algorithms depends on the protein target itself as well as its particular representation. For example, due to the possible rearrangement of binding site side chains upon ligand binding, apo forms as well as structures complexed with different molecules may yield lower docking accuracy compared to self-docking [29,30]. Therefore, using BC as a model system, we carried out benchmarking calculations for *e*SimDock [22], which is a new similarity-based docking approach, and compared the results to those using AutoDock Vina [31], which is one of the most widely used docking programs in computer-aided drug discovery. Structure-based virtual screening is essentially a large-scale cross-docking experiment, *viz.* docking of many compounds to a single ligand-bound target structure. Therefore, both algorithms, *e*SimDock and Vina, are used to dock ATP as well as a series of 13 known inhibitors to the BC structure from *E. coli* complexed with ADP (PDB-ID: 2j9g). Table 2 shows the cross-docking accuracy in terms of ligand heavy-atom RMSD from the corresponding crystal structure. Using a threshold of a 2 Å RMSD, Vina and *e*SimDock correctly reproduced binding poses of four and eight compounds, respectively. Furthermore, ATP (PDB-ID: 1dv2) and two other compounds based on the quinazoline (PDB-ID: 2w6p) and pyrimidine scaffolds (PDB-ID: 2w71) were docked by *e*SimDock with a relatively low RMSD of 3.035 Å, 2.230 Å and 3.007 Å, respectively. Thus, *e*SimDock provides a higher accuracy than Vina in the modeling of binding poses of known BC inhibitors.

**Table 2:** Accuracy of binding pose prediction for ATP and BC inhibitors using AutoDock Vina and *e*SimDock.

PDB-ID ^a^	Scaffold	AutoDock Vina ^b^	*e*SimDock ^b^
1dv2	ATP	7.443	3.035
2v58	pyrido[3,2-d]pyrimidine	2.208	0.992
2v59	pyrido[3,2-d]pyrimidine	1.372	0.980
2v5a	pyrido[3,2-d]pyrimidine	6.729	1.670
2w6m	2-amino-oxazole	7.227	0.957
2w6n	2-amino-oxazole	0.348	4.432
2w6o	7,8-dihydroquinazoline	7.038	1.331
2w6p	quinazoline	7.127	2.230
2w6q	1,3,5-triazine	6.591	0.614
2w6z	3*H*-purine	5.880	0.317
2w70	pyrimidine	5.356	1.063
2w71	pyrimidine	0.481	3.007
3jzf	1*H*-benzimidazole	0.721	5.376
3jzi	1*H*-benzimidazole	8.515	6.917

^a^ Cross-docking benchmarks were performed using BC from *E*. *coli* complexed with ADP (PDB-ID: 2j9g). ^b^ Ligand heavy-atom RMSD [Å].

### 2.3. Library of Amino-Oxazole Derivatives

**Figure 3:**
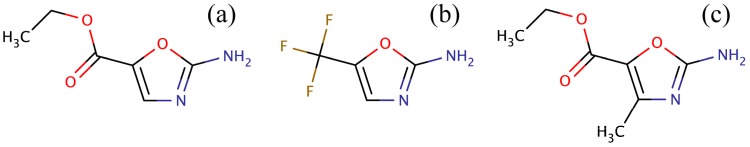
Compounds identified in the ZINC12 library by fingerprint-based virtual screening against 2-amino-oxazole. (**a**) ZINC04368839, (**b**) ZINC20357591, and (**c**) ZINC38537247.

**Figure 4:**
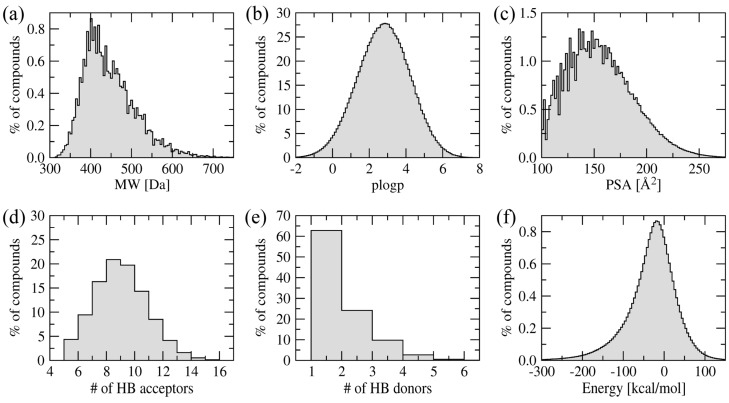
Distribution of physicochemical properties across a library of amino-oxazole derivatives. (**a**) Molecular weight, (**b**) octanol/water partitioning coefficient, (**c**) polar surface area, (**d**) the number of hydrogen bond donors and (**e**) acceptors, and (**f**) internal energy after geometry optimization in the MMFF94 force field.

Structure-based virtual screening uses molecular docking to rapidly evaluate large compound libraries against a given protein target [16,17]. Clearly, the selection of a screening library is pivotal for the success of virtual screening simulations. Searching the entire chemical space of organic compounds may be unfeasible, thus many virtual screening projects employ targeted compound libraries [32,33]. In this study, we focused on a new class of BC inhibitors based on the amino-oxazole scaffold. In order to compile a screening library, we first searched the ZINC12 collection of commercially available compounds [34] for amino-oxazole derivatives. Three compounds were identified: ZINC04368839, ZINC20357591 and ZINC38537247 (shown in Figure 3), whose Tanimoto coefficient [35] to amino-oxazole is 0.65, 0.63 and 0.55, respectively. The Tanimoto coefficient is widely used in Cheminformatics as a measure of the chemical similarity between organic compounds. It is calculated from a comparison of topological fingerprints with typical threshold values of 0.5–0.7 indicating a significant chemical similarity. Since only three compounds were identified in the ZINC database, we used virtual chemistry techniques to construct a large combinatorial library of amino-oxazole derivatives. These compounds were assembled by attaching a variety of small organic building blocks at positions R1 and R2 of the amino-oxazole scaffold (see Figure 2a). The entire collection comprises 127,751,751 molecules, ~7% of which were selected for molecular docking to BC isoforms from different bacteria species. Figure 4 shows the distribution of various physicochemical properties of the amino-oxazole derivatives. The molecular weight of the majority of compounds is within 400–500 Da. Typical values for the octanol/water partitioning coefficient and polar surface area are 1–5 and 100–200 Å^2^, respectively. Molecules in the library also have 6–12 and 1–4 hydrogen bond acceptors and donors, respectively. The vast majority of our screening compounds fit into the criteria known as the rule-of-five [36], which means they are likely to be membrane permeable and easily absorbed by the body. Furthermore, Figure 4f shows the distribution of internal energy after geometry optimization demonstrating that sterically acceptable three-dimensional representations were constructed.

### 2.4. Virtual Screening against Gram-Positive and Gram-Negative Species

Using similarity-based molecular docking techniques, the combinatorial library of amino-oxazole derivatives was subjected to structure-based virtual screening against BC isoforms from seven bacterial species including four Gram-negative and three Gram-positive organisms. The amino-oxazole substructure is assumed to adopt a similar conformation when bound to the ATP binding site of BC isoforms. This assumption is based on the observation that the amino-oxazole scaffold from two different derivatives developed by Pfizer adopts the same conformation when bound to BC from *E*. *coli* (PDB ID: 2w6m and 2w6n) [12]. Therefore, we selected 1,246,716 compounds whose amino-oxazole scaffold was consistently docked within 2 Å RMSD from that in PDB ID: 2w6n across all seven BC isoforms. Assuming the independency of individual docking calculations, we estimated from the joint probability distribution that the accuracy of ligand docking by *e*SimDock was 76%, which is in accord with docking benchmarks against BC described above as well as with the results of large-scale simulations reported previously [22].

**Figure 5:**
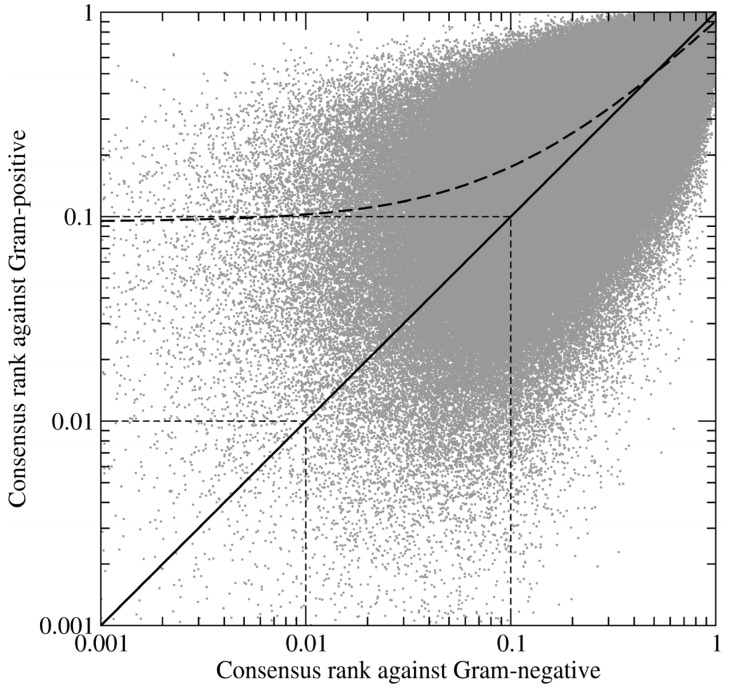
Log-log plot of the correlation between the ranks of amino-oxazole derivatives fused for Gram-positive and Gram-negative species. Each dot represents one compound; its relative ranks are expressed as the fraction of the ordered screening library. Thick solid and dashed lines are the diagonal and regression line, respectively. Thin dashed lines delineate the top 1% and 10% of the ranked library.

To maximize the ranking capabilities of our virtual screening protocol, we used data fusion (sometimes called consensus scoring), which was originally developed for applications in signal processing [37]. These techniques combine data from different sensors in order to improve the overall measurement accuracy in comparison with individual sensors. In the context of virtual screening, different sensors correspond to different scoring functions used to rank screening compounds [38,39]. Using data fusion, we first combined individual scores, the predicted binding affinity, fitness and binding scores, and the total energy of protein-ligand interactions, to rank the compounds for each BC isoform. These ranks were then fused into a consensus ranking for all BC isoforms, as well as separately against Gram-negative and Gram-positive species. Figure 5 shows the correlation between the consensus ranks of amino-oxazole derivatives against BC isoforms from Gram-positive and Gram-negative organisms. The Pearson correlation coefficient, which measures the strength of a linear association between two variables, is 0.83 with the regression line slightly shifted towards lower ranks for Gram-negative species. It suggests that compound ranking is more consistent across Gram-negative organisms, thus Gram-positive BC isoforms create a chemically diverse environment within the ATP binding site rendering the development of broad-spectrum inhibitors more difficult. This is in line with the pharmacological profiles of known BC inhibitors, which are potent against Gram-negative, but not Gram-positive species [12].

### 2.5. Profiling of ATP Binding Site

**Figure 6:**
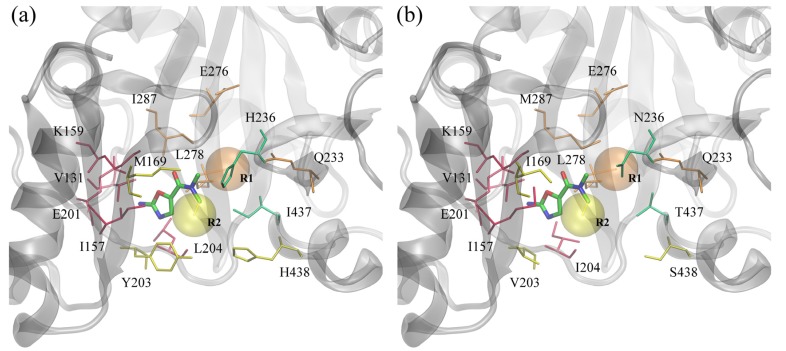
ATP binding site of biotin carboxylase from two representative organisms. (**a**) Gram-negative *E. coli* and (**b**) Gram-positive *S. pneumoniae*. Amino-oxazole fragment is shown as solid sticks colored by atom type; transparent orange and yellow spheres mark the position of two side groups R1 and R2, respectively. Selected binding residues are shown as sticks and labeled; purple, orange, yellow and green residues interact primarily with the amino-oxazole moiety, side group R1, R2, and both, respectively.

In this study, we focused on BC inhibitors that consist of three distinct parts: a fixed amino-oxazole scaffold, and variable substituents R1 and R2. The positions of these substructures within the binding pocket of BC are displayed in Figure 6. The amino-oxazole fragment interacts with residues 131, 157, 159 and 201, which are similar between Gram-negative and Gram-positive species (see Table 1). Substituents R1 and R2 interact primarily with residues 169, 203–204, 438, and 233, 276, 278, 287, respectively, whereas both moieties interact with residues 233 and 236. Many of these residues are chemically similar, e.g., M/I169, I/M287 and L/I204, however, there are some notable differences in the chemical properties of some of the amino acids between Gram-negative and Gram-positive BC isoforms, e.g., Y/V203, H/N236 and I/T437.

The structures of amino-oxazole derivatives highly ranked against all BC isoforms may give some clue on the chemical properties of side groups R1 and R2 required to target both Gram-negative and Gram-positive species. 

**Figure 7:**
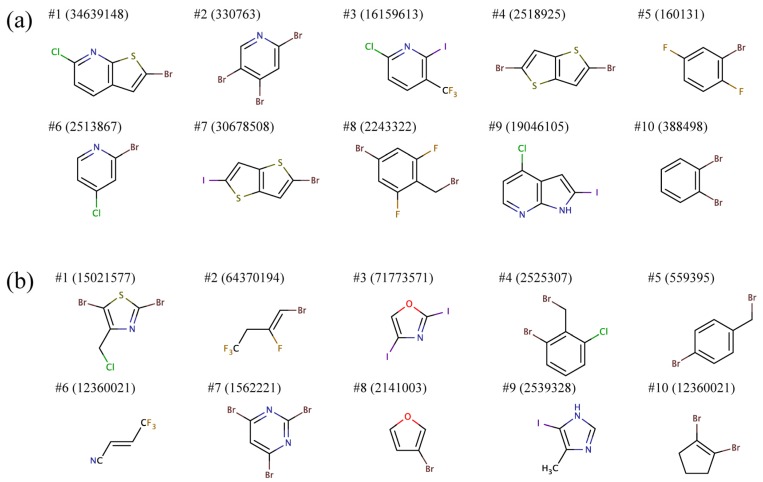
Representative chemical structures of the top ten clusters of the side groups (**a**) R1 and (**b**) R2 constructed from the top ranked amino-oxazole derivatives.

To that end, we separately clustered, based on chemical similarity, substituents R1 and R2 and selected a representative structure from each cluster. Figure 7 shows representative building blocks for the ten largest clusters for R1 and R2. Most R1 groups predicted to bind to all BC isoforms comprise mono or heterocyclic six-membered aromatic rings as well as fused five-six- and five-five-membered aromatic rings. Furthermore, these substructures typically contain two and more halogen atoms. R2 groups are predominantly composed of smaller aromatic moieties, mono or heterocyclic six- and five-membered rings, as well as short aliphatic chains. Similar to R1, R2 moieties also often include multiple halogen atoms.

**Figure 8:**
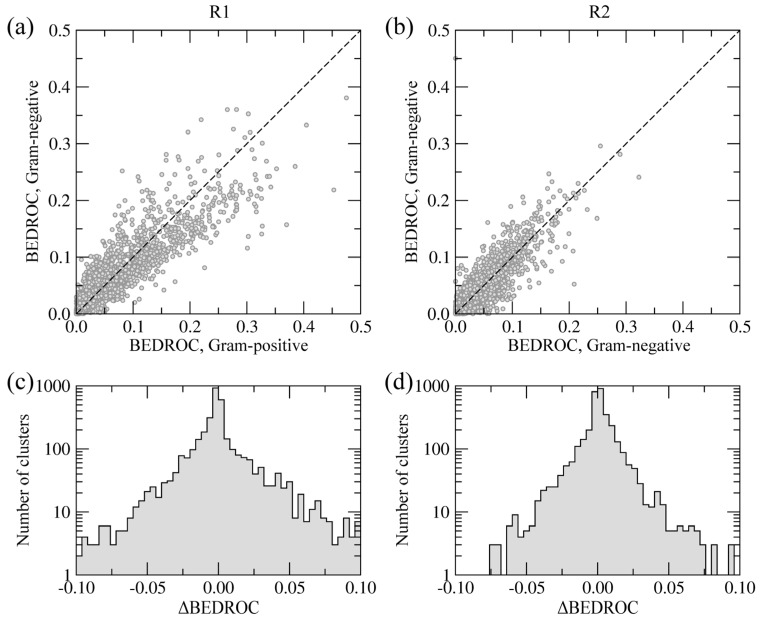
Chemical specificity of the side groups R1 (**a**, **c**) and R2 (**b**, **d**) towards Gram-positive and Gram-negative species. (**a**, **b**) Correlation between BEDROC scores calculated using the clusters of building blocks. (**c**, **d**) The distribution of ΔBEDROC values (residuals) emphasizing the differences between Gram-positive and Gram-negative BC isoforms.

In order to distinguish the similarities and differences between the side groups R1 and R2 with respect to Gram-negative and Gram-positive BC isoforms, we partitioned the library of 9,411 building blocks into 3,550 clusters using a Tanimoto coefficient threshold of 0.7. For each cluster of similar molecules, we calculated the overall enrichment at positions R1 and R2 across the screening library, for Gram-negative and Gram-positive species, separately. The BEDROC score was used to quantify the enrichment; this acronym stands for Boltzmann-enhanced discrimination of receiver operating characteristic [40]. The BEDROC score is a measure of enrichment that effectively accounts for the “early recognition problem” when analyzing ordered lists of compounds. The results are presented in Figure 8. 

Figure 8a,b show the correlation of BEDROC scores between Gram-negative and Gram-positive BC isoforms at positions R1 and R2, respectively. The Pearson correlation coefficient for R1 and R2 is 0.91 and 0.93, respectively. Furthermore, Figure 8c,d show the distribution of residuals from the correlation plots, defined as the differences between BEDROC scores for Gram-negative and Gram-positive species (ΔBEDROC). A higher frequency of ΔBEDROC values below −0.05 and above 0.05 in Figure 8c compared to Figure 8d demonstrate that more building block clusters at position R1 are ranked differently using Gram-negative and Gram-positive BC isoforms than those at position R2. Consequently, fewer chemical moieties attached at R1 have consistently high ranks against Gram-negative and Gram-positive enzymes, suggesting that this position has a potentially higher impact on the pharmacological profile of amino-oxazole derivatives.

### 2.6. Drug-Target Interactions at the Atomic Level

**Figure 9:**
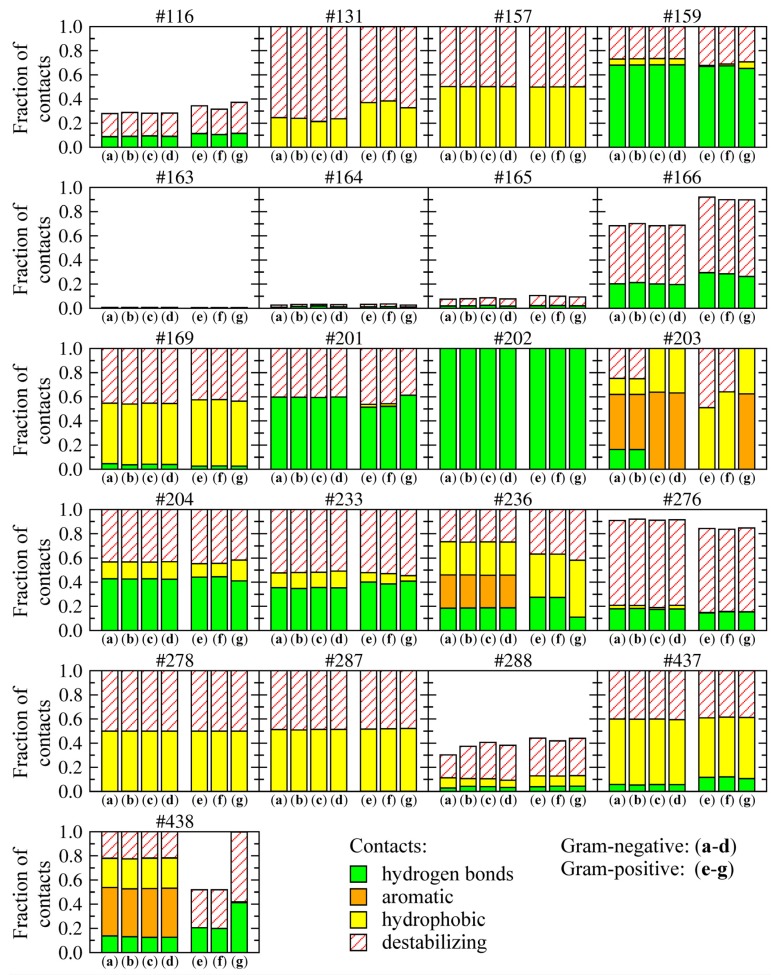
Distribution of various types of molecular interactions between the top-ranked amino-oxazole derivatives and BC isoforms from different bacteria species. Gram-negative: (**a**) *E*. *coli*, (**b**) *H*. *influenzae*, (**c**) *P*. *aeruginosa*, (**d**) *M*. *catarrhalis*; Gram-positive: (**e**) *E*. *faecalis*, (**f**) *S*. *pneumoniae*, (**g**) *S*. *aureus*. Four types of non-bonding interactions are considered: hydrogen bonds, aromatic and hydrophobic contacts, as well as hydrophilic-hydrophobic (destabilizing) contacts. Individual graphs correspond to binding residues whose numbers in the sequence are shown at the top of each graph. The height of each bar shows the fraction of compounds forming interactions with a given binding residue. The relative contribution of different interactions is shown within each bar.

A large number of constructed three-dimensional complexes between amino-oxazole derivatives and BC isoforms provide a comprehensive dataset to perform a statistical analysis of molecular drug-target interactions at the atomic level. Using 1,000 top-ranked compounds docked to each enzyme we investigated the position and frequency of various intermolecular contacts stabilizing the poses of amino-oxazole derivatives within the ATP binding site of BC. These included hydrogen bonds, aromatic and hydrophobic interactions as well as destabilizing interactions, defined as hydrophilic-hydrophobic contacts [41]. Figure 9 shows the frequency and composition of residue-level drug-target contacts. The height of each bar corresponds to the fraction of compounds forming interactions with a given residue; the relative contribution of these different interactions is shown within individual bars. For example, 18%, 28%, 27% and 27% of interactions formed between amino-oxazoles and H236 in Gram-negative species are hydrogen bonds, aromatic, hydrophobic and hydrophobic-hydrophilic contacts, respectively. In Gram-positive *E. faecalis* and *S. pneumoniae*, histidine in this position is replaced by asparagine, which interacts with 27%, 36% and 37% of compounds through hydrogen bonds, hydrophobic and hydrophobic-hydrophilic contacts, respectively.

Most binding residues interact with all top-ranked compounds, except for positions 116 and 288, which are further away, and thus, form contacts only with a subset of larger compounds, as well as the sequence of residues 163–166, which form a glycine-rich loop around the binding site. Most hydrogen bonds involve residues K159, E201, K/R202, L/I204, and Q233, which are conserved across all species and mainly interact with the amino-oxazole substructure. A number of hydrophobic contacts are formed by residues I/M157, M/I169, L/I278, I/M287, and I/T437. BC isoforms from Gram-negative species form aromatic interactions through residues Y/F203, H236, and H438, whereas equivalent residues in Gram-positive organisms form hydrogen bonds and hydrophobic interactions. The remaining contacts between hydrophobic and hydrophilic atoms are categorized as destabilizing, however, these require further investigation because one of the limitations of the algorithm used to classify protein-ligand contacts is that it designates interactions involving halogens as hydrophobic-hydrophilic. In contrast, halogen contacts in biological systems are commonly considered as weak hydrogen bonding interactions [42], thus, about half of the molecules used in drug discovery and development are halogenated. 

**Figure 10:**
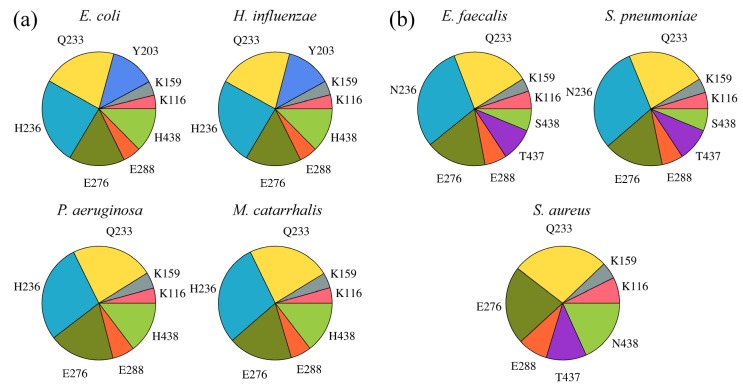
Halogen bonding pattern for amino-oxazole derivatives complexed with BC isoforms from different bacteria species. (**a**) Gram-negative, (**b**) Gram-positive. Individual pie charts show the contribution of different binding site residues to halogen bonding with the top-ranked compounds.

Since interactions involving halogens as hydrogen bond acceptors play crucial roles in the stabilization of protein-ligand complexes [43,44] and a significant number of our top-ranked compounds contain halogen atoms, we performed a separate analysis of their interaction patterns across BC isoforms. Figure 10 shows that H236 in Gram-negative and N236 in Gram-positive organisms frequently form halogen bonds with amino-oxazole derivatives, this residue position is favorably positioned to interact with both R1 and R2 side groups. Moreover, a significant number of halogen contacts involve two other residues highly conserved across all species, Q233 and E276, which primarily interact with the R1 substituent (see Figure 6). These three residues account for the majority of halogen interactions between amino-oxazole derivatives and BC enzymes. However, depending on the species-specific composition of the binding site, Y203, T437, N438 can also form halogen bonds. For instance, in the absence of a halogen bond donor at position 236 in Gram-positive *S*. *aureus*, halogenated compounds interact with E288, T437 and N438 in addition to Q233 and E276. This analysis suggests that the hydrogen bond acceptor capability of halogens can be exploited to improve the potency of amino-oxazole derivatives against Gram-positive species. 

### 2.7. Examples of Highly Ranked Compounds

**Figure 11:**
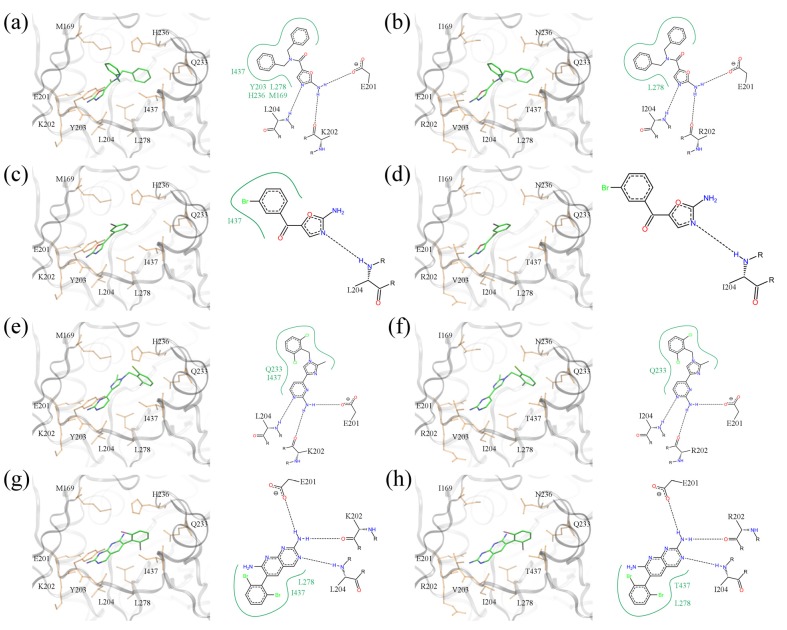
Binding poses of selected BC inhibitors. (**a**, **b**) PDB-ID: 2w6n, (**c**, **d**) PDB-ID: 2w6m, (**e**, **f**) PDB-ID: 2w71, and (**g**, **h**) PDB-ID: 2v58. BC isoforms from (**a**, **c**, **e**, **g**) *E*. *coli*, and (**b**, **d**, **f**, **h**) *S*. *pneumoniae* (*E*. *coli* residues are mutated according to *S*. *pneumoniae* sequence). In 3D representation, selected binding residues are shown as orange sticks and labeled. A schematic 2D representation of molecular interactions is shown on the right of each 3D binding site; hydrogen bonds and hydrophobic interactions are depicted as dashed black and solid green lines, respectively.

To conclude this study, we discuss the binding poses of several compounds identified by virtual screening as potential broad-spectrum BC inhibitors. But first, in order to fully comprehend why these compounds might have broad-spectrum potential, it is necessary to understand the structural basis for why the Pfizer compounds only had antibacterial activity against Gram-negative organisms. The left panel in Figure 11 shows experimentally determined three-dimensional structures of two amino-oxazole derivatives and two other halogenated compounds bound to BC from the Gram-negative *E*. *coli*. In the right panel, *E*. *coli* residues are mutated according to the sequence of Gram-positive *S*. *pneumoniae*. Figure 11a,b show a dibenzylamide prototype molecule for a series of amino-oxazole derivatives, which has two phenyl moieties at positions R1 and R2. The 2D diagram highlights three hydrogen bonds between the amino-oxazole fragment and residues 201, 202 and 204. In the *E*. *coli* isoform, both R-groups form hydrophobic contacts with residues M169, Y203, H236, L278 and I437. Because of the different amino acid composition at these residue positions in *S*. *pneumoniae*, the phenyl moieties interact only with L278. This difference may be responsible, in part, for the lack of potency of this inhibitor against Gram-positive species. The second compound shown in Figure 11c,d contains a single side group directly attached to the amino-oxazole scaffold. The bromophenyl moiety forms hydrophobic contacts with I437 in *E*. *coli*, but the 2D diagram generated using the *S*. *pneumoniae* isoform shows no direct interactions with binding site residues. The last two complexes illustrate binding poses of polyhalogenated inhibitors. 

As shown in Figure 11e–h, dichloro- and dibromophenyl moieties are located in a position close to the side group R1 of our prototype inhibitor based on the amino-oxazole scaffold. Both compounds form hydrogen bonds with residues 201, 202 and 204, which is a similar pattern as the amino-oxazole inhibitors. The compounds also interact with the side chains of Q233, L278 and I437 in the *E*. *coli* enzyme through their halogenated moieties. Thus, in Gram-positive species substituting isoleucine in position 437 with threonine may be responsible for the lower binding affinities of these BC inhibitors. This notion is supported by the fact that strains of *E. coli* that were made resistant to the amino-oxazole dibenzylamide (Figure 1b) were found to have a single mutation in which I437 was replaced with threonine [12].

**Figure 12:**
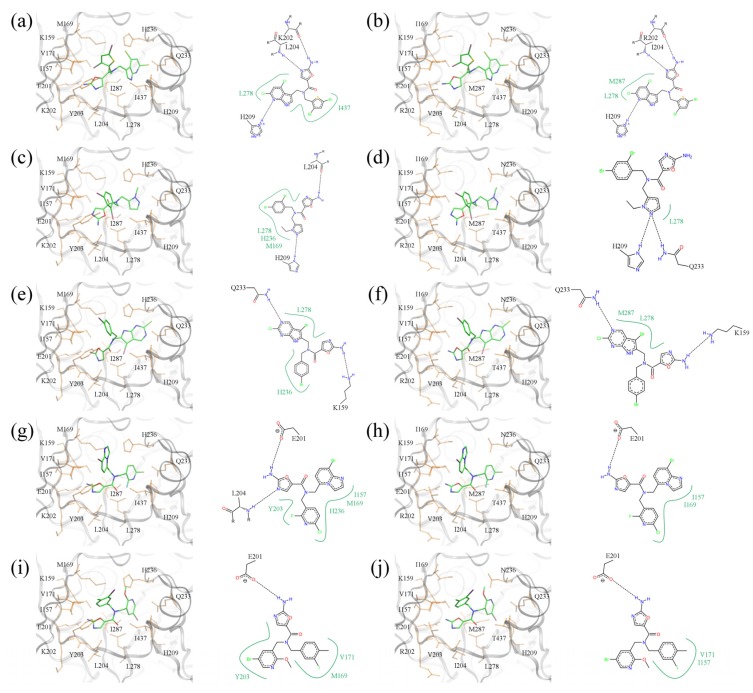
Binding poses of selected amino-oxazole derivatives docked to BC. (**a**, **b**) ao-02168779-40448781-0000, (**c**, **d**) ao-00388499-06125795-0000, (**e**, **f**) ao-00900729-40448504-0000, (**g**, **h**) ao-02149727-15042121-0000, and (**i**, **j**) ao-00403318-01672846-0002. BC isoforms from (**a**, **c**, **e**, **g**) *E*. *coli*, and (**b**, **d**, **f**, **h**) *S*. *pneumoniae* (*E*. *coli* residues are mutated according to *S*. *pneumoniae* sequence). In 3D representation, selected binding residues are shown as orange sticks and labeled. A schematic 2D representation of molecular interactions is shown on the right of each 3D binding site; hydrogen bonds and hydrophobic interactions are depicted as dashed black and solid green lines, respectively.

Five of the top-ranked amino-oxazole derivatives and their putative molecular interactions with BC from the Gram-negative *E*. *coli* (left panel) and the Gram-positive *S*. *pneumoniae* (right panel) are shown in Figure 12. In addition, the corresponding docking scores are summarized in Table 3, which shows that the amino-oxazole anchors are docked within 2 Å RMSD from that in the crystal structure of BC complexed with a known amino-oxazole inhibitor. Fitness and binding probability scores reported by *e*SimDock are close to 1 suggesting that there are no steric clashes and the compounds form favorable interactions with the enzymes; this is also supported by the all-atom interaction energy calculated using AMMOS. Moreover, the predicted binding affinities are in the nanomolar range. In most modeled complexes, binding of the amino-oxazole scaffold is stabilized by multiple hydrogen bonds with the side chain carboxylic acid of E201, and the backbone nitrogens of K202 and L204, which is consistent with available crystal structures of BC complexed with inhibitors. The first compound shown in Figure 12a,b contains both polyhalogenated side groups and interacts with residues L278, I437, and L278, M287 in the *E. coli* and *S*. *pneumoniae* enzymes, respectively. Interestingly, a larger pyrrolopyridine moiety attached at position R1 reaches deeper into the binding site to form a hydrogen bond with H209 in both isoforms. The second compound shown in Figure 12c,d revealed a similar interaction with H209 through its pyrazole moiety attached at R1, whereas the dihalogenated phenyl substituent at R2 interacted with L278 in BC from both Gram-positive and Gram-negative organisms. The compound shown in Figure 12e,f contains a halogenated pyrrolopyrimidine moiety at R1, which interacts with the side chain amide of Q233 as well as with the hydrophobic side chain of L278; these interactions are present in both BC isoforms. Halogenated aromatic substituents of the last two compounds in Figure 12g–j make extensive contacts with a hydrophobic pocket formed by residues at positions 157, 169, 171 and 203. These examples highlight potential interactions that could be exploited in order to increase the potency of amino-oxazole inhibitors towards BC enzymes from both Gram-negative as well as Gram-positive species.

## 3. Experimental

### 3.1. Construction of the Screening Library

A library of low molecular weight organic building blocks was compiled from the catalogues of the following 20 vendors included in the ZINC12 library [45]: ACB Blocks, Accela ChemBio, Angene Building Blocks, Aronis BB, Asinex Building Blocks, AsisChem Building Blocks, BePharm Building Blocks, ChemDiv BuildingBlocks, ChemBridge BuildingBlocks, Combi-Blocks, EvoBlocks, Life Chemicals BB, Key Organics Building Blocks, Maybridge Building Blocks, Sigma Aldrich Building Blocks, SynQuest Building Blocks, Synthonix Building Blocks, Tetrahedron Building Blocks, TimTec BB, and Zylexa Pharma BB. We included only fragments that have 6–12 heavy atoms. Removing the redundancy at a Tanimoto coefficient [35] threshold of 0.95 using the SUBSET program [46] resulted in 9,411 compounds. Next, all combinations of two building blocks that together have up to 18 heavy atoms were attached at positions R1 and R2 of the amino-oxazole scaffold shown in Figure 2a. Each building block was coupled to the amide nitrogen of amino-oxazole through a hydrogenated carbon, nitrogen, oxygen or sulfur atom. All possible combinations were explored, so a pair of two building blocks could result in a series of chemically different compounds. Finally, using SUBSET, the redundancy was removed at a Tanimoto coefficient of 0.95; the constructed library of amino-oxazole derivatives consists of 127,751,751 different molecules.

**Table 3:** Examples of top ranked amino-oxazole derivatives selected by virtual screening against BC and their docking scores.

Compound ID ^a^	ao-RMSD ^b^ [Å]		Fitness ^c^		Binding ^d^		Affinity ^e^		Energy ^f^ [kcal/mol]	
	*Ec* ^g^	*Sp* ^g^	*Ec*	*Sp*	*Ec*	*Sp*	*Ec*	*Sp*	*Ec*	*Sp*
ao-02168779-40448781-000	1.41	1.42	1.00	1.00	1.00	1.00	0.71	0.79	38.19	50.52
ao-00388499-06125795-000	1.79	1.84	1.00	1.00	1.00	0.91	0.36	-0.05	40.68	4.30
ao-00900729-40448504-000	1.93	1.92	1.00	1.00	0.99	0.96	−0.12	0.55	37.96	20.95
ao-02149727-15042121-000	1.81	1.82	1.00	1.00	1.00	0.99	0.22	0.57	84.23	32.37
ao-00403318-01672846-002	1.83	1.83	1.00	1.00	1.00	0.99	−0.45	0.39	89.74	34.06

^a^ the first field is a code for amino-oxazole, the second and third fields are the ZINC-ID of R1 and R2, respectively, the last field is a combination number. ^b^ All-atom RMSD calculated over the amino-oxazole fragment *vs.* 2w6n. ^c^ Fitness and ^d^ binding scores reported by *e*SimDock. ^e^ Binding affinity predicted by *e*SimDock, expressed as ln *K*_i_. ^f^ Interaction energy reported by AMMOS. ^g^ Ec — *E*. *coli*, Sp — *S*. *pneumoniae*.

### 3.2. Generation of 3D Conformations and Conformational Ensembles

We used Open Babel [47] to generate three-dimensional molecular structures for a subset of 10 × 10^6^ compounds randomly selected from the library of amino-oxazole derivatives, followed by geometry optimization in the Merck MMFF94 force field [48]. This procedure resulted in 8,898,942 “clean” chemical structures without atomic clashes and having acceptable bond lengths and angles. Subsequently, for each compound, we generated a large ensemble of low-energy conformations using Balloon and the MMFF94-like force field [49]. An initial ensemble was subject to the clustering procedure using CLUTO [50] and a pairwise similarity threshold of 1 Å RMSD for the atomic coordinates. Depending on the number of rotatable bonds, the final non-redundant ensemble contains up to 50 different low-energy structures.

### 3.3. BC Isoform Structures

The crystal structure of BC from *Escherichia coli* (PDB-ID: 2j9g) was used to model BC isoforms from the following bacteria species: (Gram-negative) *Haemophilus influenzae*, *Pseudomonas aeruginosa*, *Moraxella catarrhalis*, (Gram-positive) *Enterococcus faecalis*, *Streptococcus pneumoniae*, and *Staphylococcus aureus*. Side chains of binding site residues were mutated to those from a different species according to multiple sequence alignments reported previously [23] and the resulting structures were subjected to energy minimization. Both residue mutation and energy optimization was carried out using the Jackal modeling package [51]. The assignment of partial charges to target protein structures as well as the conversion to the PDBQT format were done using MGL Tools [52].

### 3.4. Similarity-Based Ligand Docking and Pose Refinement

Amino-oxazole derivatives were docked to all seven BC isoforms using the recently developed *e*SimDock [22]. The ability of *e*SimDock to predict ligand binding poses and the corresponding affinities was previously benchmarked on a large dataset of 1,151 complexes selected from BindingDB [53] using crystal structures as well as the modeled conformations of target proteins [22]. Furthermore, these benchmarking simulations included two case studies on the inhibitors of fXa and CDK2, which are computationally similar to docking of amino-oxazole derivatives to BC isoforms. Using *e*SimDock gave equally encouraging results in both cases, therefore we selected this approach for the current study focusing on BC inhibitors. As the anchor, we used the conformation of amino-oxazole substructure from the crystal structure of BC complexed with 2-amino-*N*,*N*-bis(phenylmethyl)-1,3-oxazole-5-carboxamide (PDB-ID: 2w6n). *e*SimDock performs a rigid-body superposition of each conformation from the docking ensemble onto the anchor substructure and optimizes the orientation of flexible regions (in our case the side groups R1 and R2) within the binding site of the target structure. Next, the best binding pose was selected using non-linear machine learning models; the final docking result was also assigned a binding score and binding affinity. In addition, ligand poses modeled by *e*SimDock were refined by molecular mechanics using AMMOS [54] and the standard AMMP force field sp5 [55].

### 3.5. Ligand Scoring and Ranking

For each amino-oxazole derivative docked into each BC isoform, we calculated the heavy-atom RMSD from the amino-oxazole anchor substructure (PDB-ID: 2w6n). Next, we selected only those conformations whose RMSD is ≤2 Å across all BC complexes; this procedure resulted in 1,246,716 compounds. Ligand ranking was performed using data fusion with the SUM rule [37,56] and the following 4 docking scores: (from *e*SimDock) predicted binding affinity, predicted fitness and binding scores, (from AMMOS) the total energy of protein-ligand interactions. Three rank-ordered lists of compounds were constructed separately for Gram-negative and Gram-positive organisms, as well as for all species.

### 3.6. Analysis of Drug-Target Interactions

Our primary tool for analyzing protein-ligand interaction is LPC (Ligand-Protein Contacts), which detects and classifies interatomic contacts based on the solvent-accessible surface calculations and evaluates the interface complementarity [41]. In addition, binding poses of amino-oxazole derivatives in BC pockets were visualized in 3D using VMD (Visual Molecular Dynamics) [57] and flattened to 2D complex diagrams by PoseView [58].

## 4. Conclusions

In an effort to improve the pharmacological profile of biotin carboxylase inhibitors, we performed virtual screening of a diverse combinatorial library of amino-oxazoles against isoforms from several Gram-negative and Gram-positive bacteria species. The amino-oxazole scaffold was selected primarily because of its high synthetic tractability, *viz*. the carboxyl group on amino-oxazole can be easily coupled to fragments containing a nucleophilic nitrogen using standard peptide chemistry to generate novel series of compounds. Structure-based virtual screening was conducted using *e*SimDock, a new similarity-based docking approach. Under the assumption that the amino-oxazole substructure adopts a similar conformation when complexed with different BC isoforms, the accuracy of ligand docking by *e*SimDock is 76%. By analyzing compound ranking, we find that the development of broad-spectrum inhibitors is challenging because the ATP binding site of BC creates a chemically diverse environment, particularly across Gram-positive species. Amino-oxazole derivatives contain two side groups, R1 and R2. Most R1 groups in inhibitors selected by virtual screening comprise either mono or heterocyclic, six-membered or fused aromatic rings, whereas smaller aromatic moieties and short aliphatic chains are attached at R2. Moreover, both the R1 and R2 side groups are frequently halogenated. Comparing side groups R1 and R2 across Gram-negative and Gram-positive BC isoforms indicates that R1 may have a potentially higher impact on the pharmacological profile of amino-oxazole derivatives. The analysis of binding poses of the top-ranked compounds suggests that (1) binding of the amino-oxazole anchor is stabilized by a network of hydrogen bonds to residues 201, 202 and 204; (2) halogenated aromatic moieties enhance interactions with a hydrophobic pocket formed by residues 157, 169, 171 and 203; and (3) larger moieties attached at R1 reach deeper into the binding pocket to form hydrogen bonds with the side chains of conserved residues 209 and 233. These insights obtained from the present *in silico* study will be next tested experimentally in both *in vitro* and *in vivo* systems.
